# LSVT® BIG versus progressive structured mobility training through synchronous telerehabilitation in Parkinson’s disease: A randomized controlled trial

**DOI:** 10.1007/s10072-024-07322-0

**Published:** 2024-01-25

**Authors:** Guzin Kaya Aytutuldu, Burcu Ersoz Huseyinsinoglu, Nazan Karagoz Sakalli, Aysu Sen, Ipek Yeldan

**Affiliations:** 1 Department of Physiotherapy and Rehabilitation (English), Faculty of Health Sciences, Biruni University, Istanbul, Turkey; 2 Institute of Graduate Studies, Istanbul University-Cerrahpasa, Istanbul, Turkey; 3 Department of Physiotherapy and Rehabilitation, Faculty of Health Sciences, Marmara University, Istanbul, Turkey; 4 Department of Neurology, Bakırkoy Research and Training Hospital for Neurologic and Psychiatric Diseases, Istanbul, Turkey; 5 Present Address: Department of Physiotherapy and Rehabilitation, Faculty of Health Sciences, Istanbul University-Cerrahpasa, Istanbul, Turkey

**Keywords:** LSVT® BIG, Parkinson’s disease, Physiotherapy, Postural control, Telerehabilitation

## Abstract

**Background:**

Parkinson’s disease (PD) is a common neurodegenerative illness associated with motor symptoms.

**Aim:**

The aim of study was to compare the effects of synchronous telerehabilitation-based Lee Silverman Voice Treatment**®** BIG (LSVT® BIG) protocol and progressive structured mobility training in patients with Parkinson’s disease (PD).

**Methods:**

Thirty-two patients diagnosed with PD (aged 40–72 years, Hoehn-Yahr stage 1–3) were randomly allocated into LSVT® BIG (Group 1) and Progressive Structured Mobility Training (Group 2) groups. Exercises were performed in both groups for 60 min a day, 4 days a week, for 4 weeks under the supervision of a physiotherapist with synchronous online videoconference method. Dynamic balance was assessed with Mini-Balance Evaluation Systems Test (Mini-BESTest) as a primary outcome measure. The secondary outcome measurements were Timed Up and Go Test (TUG), spatiotemporal parameters of gait from Kinovea® software, and postural stability from the Biodex Balance System. Other outcome measures were Activity-Specific Balance Confidence Scale-Short Form (ABC-SF), Parkinson’s Activity Scale (PAS), and Parkinson’s Disease Quality of Life Questionnaire (PDQ-39).

**Results:**

This study showed significant group-by-time interactions on Mini-BEST (*p* = 0.042), ABC-SF (*p* = 0.029), and PAS (*p* = 0.022) in favor of group 1. Also, TUG (*p* < 0.01), spatiotemporal parameters of gait (*p* < 0.01), and PDQ-39 (*p* < 0.01) were improved in both groups.

**Conclusion:**

Both synchronous telerehabilitation-based exercise protocols enhanced balance and gait, as well as activity level and quality of life in patients with PD. LSVT® BIG may be preferred to improve dynamic balance, balance confidence, and activity status in the early stages of PD. These results should be confirmed in future studies with more robust methodology.

**Trial registration:**

NCT04694872.

**Supplementary Information:**

The online version contains supplementary material available at 10.1007/s10072-024-07322-0.

## Introduction

Parkinson’s disease (PD) is a degenerative process that primarily affects the brainstem’s substantia nigra and other pigmented neurons. The clinical symptoms are resting tremor, bradykinesia, rigidity, and postural dysfunctions [1]. Gait disorders are quite common in PD, and the most common impairments during walking are decreased arm swing, stride length, speed, stride width, and postural control inadequacy [2]. Gait disorders are also closely related to the severity of the disease, and with the progression of the disease, the frequency of falls increases due to insufficient balance control. Different exercise approaches have been suggested for the treatment of balance and gait problems in these patients. One of the current approaches is the Lee Silverman Voice Treatment**®** BIG (LSVT**® **BIG) which is a high-intensity exercise protocol. The intensity of exercise in the treatment of LSVT**®** BIG is suggested as 16 individual training sessions of 1 h, 4 times a week for 4 weeks [3]. This approach consists of large-amplitude exercises and aims to restore the normal amplitude of movement by re-adjusting the patient’s perception of movement and walking [4].

Although the evidence for the efficacy of LSVT® BIG therapy is limited [5], the European Physiotherapy PD Guidelines recommend this exercise approach to improve walking, balance, transfers, and physical capacity [6]. In this guide, it is noted that exercise programs that include balance activities also play an important role in the rehabilitation of PD. However, studies comparing the efficacy of exercise types and application methods conducted at a similar intensity with LSVT® BIG are limited in the literature [7–9].

The applicability of the face-to-face treatment in the clinical environment has temporal restriction and brings economic burden especially in chronic neurological diseases such as PD. The COVID-19 pandemic process also has showed the importance of remote health services to reduce the risk of transmission [10]. For some time, telerehabilitation or remote rehabilitation interventions have been used to overcome the identified barriers and to increase compliance with exercise in the PD population [10, 11]. However, there is no consensus on the most effective content of exercise program to improve balance and mobility dysfunction of patients with PD for both the clinical environment and remote settings. It is known that LSVT® BIG is a program with a high frequency of exercise aimed at increasing the amplitude of motion. However, it is not known whether the improvement obtained from the LSVT® BIG protocol in early mid-stage PD is due to the exercise intensity or the content focused on amplitude enhancement. Also, there is limited evidence in the literature to support the implementation of intensive exercise programs such as LSVT® BIG through telerehabilitation [12]. The purpose of this study was to compare the effects of two different exercise protocols with the same dosage delivered through synchronous telerehabilitation on balance and mobility dysfunction in patients with PD. It primarily aimed to investigate and compare the effects of synchronous telerehabilitation-based LSVT® BIG treatment and progressive structured mobility training on dynamic balance in Parkinson’s patients. Our secondary aim was to compare the effects of two different exercise programs given with synchronous telerehabilitation on functional mobility, gait parameters, and postural stability.

## Methods

### Study design

This study is a single blinded, randomized clinical study collectively conducted by Bakırkoy Research and Training Hospital for Neurologic and Psychiatric Diseases and the ……………………………… Istanbul University-Cerrahpasa, Department of Physiotherapy and Rehabilitation. The study group included 40–75 years old with PD who were admitted to the outpatient neurology department. Ethical approval for this study was obtained from the Istanbul University-Cerrahpasa, Faculty of Medicine Clinical Research Ethics Committee (Approval number: A-03/2020) and registered as clinicaltrials.gov (NCT04694872). Informed consent was obtained from the patients. This study was performed in compliance with the Declaration of Helsinki.

The sample size and power calculations were performed with G*Power 3.1 power analysis program. The number of participants that should be included was calculated as 30 in total (15 for each group) with the difference of 3.4 (Minimal Clinically Important Difference value for the Mini Balance Evaluation System Test (Mini-BEST) test for Parkinson’s disease, standard deviation: 3.1) [13] and 80% power (type 1 error = 0.05). Assuming 15% dropout rate, we recruited 34 subjects into the study. The 34 patients who met the inclusion criteria were randomized into two groups using the “Research Randomiser” website which is an online randomization web service (https://www.randomizer.org/).

Participants whose diagnosis of idiopathic Parkinson’s disease was confirmed by a neurologist and who met the inclusion criteria were included in the study. Participants were included in the study if their diagnosis of idiopathic PD was confirmed by a neurologist, and they met the inclusion criteria. Inclusion criteria for the study were stage 1–3 according to the Hoehn and Yahr staging system, at least 21 points on the Montreal Cognitive Assessment, stable drug treatment for the past month, and the ability to walk independently on flat ground (score of 3 or more on the Functional Ambulation Classification). Individuals who had hearing or vision problems; neurological, cardiovascular, or orthopedic disorders that may prevent walking; and did not have internet access with a computer were not included. The clinical conditions of patients with PD were followed by same two neurologists during the study.

### Outcomes

Measurements were completed in the clinical setting before interventions began and after the 4-week treatment program was completed. The demographic and disease-related features were recorded for each patient. All the evaluations and interventions were performed by the same physiotherapist who had certification in LSVT® BIG protocol (GKA).

#### Primary outcome measure

The Mini Balance Evaluation System Test (Mini-BEST) balance scale was performed as a primary outcome measure was used in the evaluation of dynamic balance in our study. This scale is a 14-item, one-dimensional and extremely reliable measurement method that takes approximately 15 min to complete [14]. Mini-BESTest reveals balance problems by focusing on postural responses, dynamic gait, and sensory orientation and is frequently used in patients with PD. Each item is scored between 0 and 2. Zero points indicate that the person does not fulfill that task; 28 points is the maximum score that can be obtained from the test and is the best indicator. [14].

#### Secondary outcome measures

The secondary outcome measurements were (1) Timed Up and Go Test, (2) gait parameters from Kinovea, and (3) postural stability scores from the Biodex Balance System.

The Timed Up and Go Test (TUG) is a simple, widely used, and rapid test for assessing mobility, balance, and fall risk in patients with PD [15]. To perform the test, participants must get up from a standard chair, walk comfortably to a place 3 m off the ground, turn around and return to the chair, and sit in the same chair. In our study, all steps of the test were measured in seconds using a stopwatch.

The participants’ spatiotemporal parameters of gait were collected by using Kinovea® software which is used to perform linear and/or angular kinematic analysis of movements through videos collected using different tools. It can be used to obtain objective and quantitative data for the spatiotemporal parameters of gait [16]. While the Kinovea® version 0.8.15 software was used for data analysis of movements, it was also used to obtain spatiotemporal parameters of gait in Parkinson’s patients [17]. In our study, the 3-m walking distance of the cases was recorded with a camera placed on the sagittal plane. Colored signs placed on the right and left heels were marked on the video. By observing the videos obtained, the marks of the right and left foot heels were specified on the computer, and the double stride length was calculated with Kinovea motion analysis software. Several gait cycles were recorded in the video to express cadence and double stride lengths [18, 19]. Velocity (V) was calculated considering stride length and stride time using the following formula: *V* = distance (m)/ time (s).

The Biodex Balance System (BBS; Biodex Medical System Inc., Shirley, NY, USA) was used to measure the displacement of the body center of gravity (COG). The BBS consists of a circular platform that allows 20° of platform tilt in 360° of range of motion [20]. The measures of postural stability scores for BBS include The General Sway Index, Medial–Lateral Sway Index, and Anterior–Posterior Sway Index. The degree of instability of the surface platform can be adjusted from the most stable level (Level 8) to the least stable level (Level 1). In our study, the surface was adjusted to be at the most stable level (Level 8).

#### Other outcome measures

The other outcome measures were (1) Activity-Specific Balance Confidence Scale (ABC-SF), (2) Parkinson’s Activity Scale (PAS), and (3) The Parkinson’s Disease Quality of Life Questionnaire (PDQ-39).

The Short Form of the Activity-Specific Balance Confidence Scale (ABC-SF) was used to measure the balance confidence of the patients. The ABC consists of 16 items and is answered with the self-perception of the individual. It contains values in the range of 0–100 and measures the perceived ability to maintain balance under different conditions. The highest score indicates full confidence in balance abilities [21].

Parkinson’s Activity Scale (PAS), a scale is developed to evaluate functional activities in PD, provides information about the transfer status of patients. Scored between 0 and 4 points in this scale, a high score indicates good performance. The scale has subsections such as getting up from a chair, in-bed mobilization, and walking akinesia [22, 23].

The Parkinson’s Disease Quality of Life Questionnaire (PDQ-39) was used to evaluate the quality of life. PDQ-39 is a self-report questionnaire that uses a 5-point Likert scale to evaluate quality of life such as the severity of symptoms of mobility, activities of daily living, emotional well-being, social support, cognition, and communication. A higher score indicates a lower quality of life [24].

### Interventions

At the start of the program, each participant explained the program to which they had been assigned, and the treatment started the same week. The participants who met included criteria were randomized to group 1 (LSVT**®** BIG Protocol Group) and group 2 (Progressive Structured Mobility Training Group). In both groups, the exercises were carried out 4 days a week, 60 min a day, for 4 weeks, using real-time videoconferencing (Zoom) under the supervision of a physiotherapist.

*Group 1* performed the LSVT® BIG protocol based on synchronized telerehabilitation. LSVT**® **BIG is a protocol derived from LSVT**® **LOUD, used in neurorehabilitation, consisting of large amplitude functional movements [9]. Maximal daily exercises consist of seven standard exercises. Functional component tasks are created for the movements that the patient has difficulty in daily life activities, which are selected individually based on the patient’s complaints. Daily living activities also take part in hierarchical tasks with large amplitudes. Participants were asked to fill in the functional task component registration form during the evaluation to determine the functional component tasks and hierarchical tasks. A personalized program was created by considering the daily life activities in this form, which the subjects had difficulty in. The participants were given gait training, and the treatment program included gait training focusing on taking big steps with reciprocal arm swings (Supplement 1).

*Group 2* consisted of structured mobility-oriented exercises such as reaching, taking a step back and forth, weight transfer, counting, sit-stand exercise, walking, changing direction, or walking over obstacles. The exercise program was followed by completing cognitive secondary tasks such as counting numbers and alphabets in the third week and motor secondary tasks of opening and closing the fists in the fourth week (Supplement 2).

### Statistical analysis

Statistical program of “Statistical Package for Social Sciences” (SPSS) version 21.0 (SPSS Inc., Chicago, IL, USA) was used for statistical analysis of study data. In all analyses, *p* < 0.05 (two-sided) values were considered statistically significant. Whether the data were suitable for normal distribution was determined with the “Shapiro Wilk Test.” The groups were compared in terms of demographic, clinical characteristics, and baseline variables with the “Independent Sample T” test or the “Chi-square” test. “Paired Sample T” test was used to compare the values of the groups before and after the treatment. The effect size was calculated using the formula (EB) = difference between measurements / standard deviation of the first measurement. The effect size was interpreted as 0.20–0.50 “small”, 0.51–0.80 “medium”, and 0.81 and above as “large”. Repeated measure ANOVA (2 × 2) analysis was used to analyze the group-by-time interactions on outcomes. This analysis was assessed by calculating the intention-to-treat (ITT) effect in the data with two dropout cases.

## Results

**Fig. 1:**
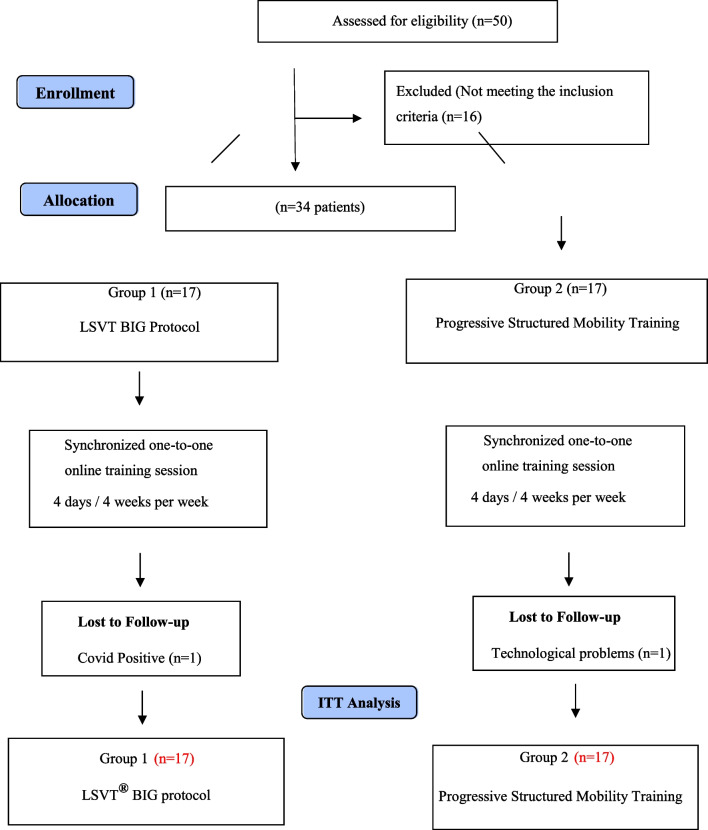
Flowchart of the study

Sixteen of the 50 volunteers did not meet the inclusion criteria and were excluded from the study. The remaining 34 subjects were randomized into 2 groups. One person from group 1 withdrew from the study due to being covid positive, and one person from group 2 withdrew from the study due to technical problems. The 4-week training was completed with a total of 32 patients with PD (Fig. 1). Thirty-four subjects were included in the ITT analysis. Analysis of the data on an ITT basis, with missing cases replaced by the mean values for each parameter obtained from participants who attended the final assessment, gave similar results.

There were no statistical differences between the groups in terms of demographic and disease-related characteristics of the participants at baseline. Also, all balance and gait parameters’ scores were similar, as shown in Table 1.

**Table 1:** Comparison of the Groups at Baseline

	Group 1 (*n* = 17) mean ± SD / *n* (%)	Group 2 (*n* = 17) mean ± SD/ *n* (%)	*p* values
Age (years)	58.47 ± 8.76	61.17 ± 6.93	0.32
Sex			
Female	4 (25)	4 (25)	1.00
Male	12 (75)	12 (75)	
BMI (kg/m^2^)	27.93 ± 2.94	26.56 ± 3.92	0.25
Disease duration (years)	4.82 ± 3.79	6.64 ± 4.24	0.19
UPDRS-III (score)	22.47 ± 5.94	27.94 ± 11.89	0.10
LED (mg)	761.58 ± 404.63	819.58 ± 440.45	0.69
Hoehn and Yahr Stages	2.08 ± 0.50	2.02 ± 0.48	0.73
Mini-BEST	19.47 ± 2.74	19.05 ± 3.61	0.71
TUG	9.03 ± 2.11	10.31 ± 5.35	0.36
AP Sway Index	0.52 ± 0.23	0.53 ± 0.22	0.88
ML Sway Index	0.48 ± 0.38	0.48 ± 0.23	0.98
General Sway Index	0.73 ± 0.40	0.71 ± 0.31	0.86
Gait Speed (m/sn)	1.17 ± 0.22	1.30 ± 0.35	0.20
Double Step Length (cm)	141.59 ± 22.60	148.46 ± 29.58	0.45
Cadence	101.64 ± 21.98	105.17 ± 14.59	0.58
ABC-SF (%)	57.88 ± 16.97	59.84 ± 24.06	0.78
PAS	31.05 ± 2.41	31.0 ± 5.47	0.96
PDQ-39	34.07 ± 12.55	39.47 ± 18.19	0.32

Group 1, LSVT® BIG Protocol; group 2, Progressive Structured Mobility Training

*SD* standard deviation, *UPDRS-III* Unified Parkinson’s Disease Rating Scale III, *LED* levodopa equivalent dose (mg), *TUG* Timed Up and Go Test, *sec* second, *AP* anteroposterior, *ML* mediolateral, *ABC-SF* Activity of Balance Confidence Short Form, *PAS* Parkinson Activity Scale, *PDQ-39* Parkinson’s Disease Questionnaire-39

^*^p < 0.05; **p < 0.01

Mobility and balance parameters were presented as the mean and standard deviation in Table 2. At the end of 4 weeks, the interaction effects (time × group) revealed a significant difference for Mini-BEST (*F* = 4.32, *p* = 0.04) in favor of LSVT® BIG group.

**Table 2:** Comparison of mobility and balance parameters of the participants within and between groups

	Baseline mean ± SD	After treatment mean ± SD	Within group change mean [CI]	Effect Size	Time × group		
					*p* value	*F*	ηp2
Mini-BEST Test							
Group 1	19.47 ± 2.74	24.24 ± 2.73	4.77 [3.74–5.83]**	1.74	0.04	4.32	0.119
Group 2	19.05 ± 3.61	22.49 ± 3.30	3.43 [2.70–4.22]**	0.95			
TUG (sec)							
Group 1	9.03 ± 2.11	7.00 ± 1.92	− 2.02 [− 2.67– − 1.31]**	0.96	0.249	1.378	0.041
Group 2	10.31 ± 5.35	7.07 ± 1.93	− 3.23 [− 5.29– − 1.75]*	0.6			
AP Sway Index							
Group 1	0.52 ± 0.23	0.38 ± 0.10	− 0.14 [− 0.23– − 0.05]*	0.6	0.247	1.392	0.042
Group 2	0.53 ± 0.22	0.47 ± 0.18	− 0.06 [− 0.16–0.01]	0.27			
ML Sway Index							
Group 1	0.48 ± 0.38	0.28 ± 0.16	− 0.19 [− 0.40– − 0.06]*	0.52	0.141	2.276	0.066
Group 2	0.48 ± 0.23	0.43 ± 0.25	− 0.05 [− 0.13–0.01]	0.21			
General Sway Index							
Group 1	0.73 ± 0.40	0.47 ± 0.18	− 0.26 [− 0.46– − 0.11]*	0.65	0.086	3.146	0.09
Group 2	0.71 ± 0.31	0.64 ± 0.29	− 0.06 [− 0.20–0.03]	0.22			

Group 1, LSVT® BIG (Protocol; Group 2, Progressive Structured Mobility Training

*TUG* Timed Up and Go Test, *sec* second, *SD* standard deviation, *AP* anteroposterior, *ML* mediolateral, *CI* confidence interval, *ηp2* partial eta

^*^p < 0.05; **p < 0.01

Both groups showed significant improvement in terms of TUG, AP sway, ML sway, and general sway within group changes (*p* < 0.05). Gait parameters were presented as the mean and standard deviation in Table 3. Both groups showed significant improvement in terms of gait speed and double step length within group changes (*p* < 0.05) after 4 weeks of treatment. However, there was no significant group × time interaction for the variables resulting from these objective measurements.

**Table 3:** Comparison of gait parameter results of the participants within and between groups

	Baseline mean ± SD	After treatment mean ± SD	Within group change mean [CI]	Effect Size	Time × group		
					*p* value	*F*	ηp2
Gait speed (m/sn)							
Group 1	1.17 ± 0.22	1.44 ± 0.28	0.26 [0.17–0.37]**	1.22	0.838	0.042	0.001
Group 2	1.30 ± 0.35	1.55 ± 0.35	0.25 [0.15–0.36]**	0.71			
Double step length (cm)							
Group 1	141.59 ± 22.60	177.12 ± 31.01	35.62 [22.58–51.77]**	1.57	0.152	2.153	0.063
Group 2	148.46 ± 29.58	169.96 ± 33.41	21.50 [11.43–34.54]*	0.72			
Cadence							
Group 1	101.64 ± 21.98	98.38 ± 17.50	− 3.26 [− 11.23–3.14]	0.14	0.114	2.644	0.076
Group 2	105.17 ± 14.59	110 ± 12.31	4.82 [− 1.28–11.87]	0.33			

Group 1, LSVT® BIG Protocol; group 2, Progressive Structured Mobility Training

*SD* standard deviation, *CI* confidence interval, *ηp2* partial eta

^*^p < 0.05; **p < 0.01

The activity balance confidence, activity status, and PDQ-39 results were shown in Table 4. There were significant differences in group by time interactions for ABC-SF (*F* = 6.963, *p* = 0.013) and PAS (*F* = 6.691, *p* = 0.014) from these self-rated evaluations in favor of the LSVT® BIG group.

**Table 4:** Comparison of balance confidence, activity statu, and PDQ-39 results of the participants within and between groups

	Baseline mean ± SD	After treatment mean ± SD	Within group change mean [%95 CI]	Effect size	Time × group		
					*p* value	*F*	ηp2
**ABC-SF (%)**							
Group 1	57.88 ± 16.97	77.61 ± 14.73	19.37 [16.06–22.68]**	1.16	0.013*	6.963	0.179
Group 2	59.84 ± 24.06	70.93 ± 22.32	11.09 [6.75–16.62]**	0.46			
**PAS**							
Group 1	31.05 ± 2.41	36.23 ± 1.67	5.17 [4.29–6.11]**	2.14	0.014*	6.691	0.173
Group 2	31.0 ± 5.47	34.41 ± 4.19	3.41 [2.47–4.41]**	0.62			
**PDQ-39**							
Group 1	34.07 ± 12.55	16.78 ± 10.12	− 17.28 [− 21.09– − 13.67]**	1.37	0.457	0.567	0.017
Group 2	39.47 ± 18.19	24.55 ± 12.37	− 14.92 [− 19.84– − 10.70]**	0.82			

Group 1, LSVT® BIG Protocol; group 2, Progressive Structured Mobility Training

*ABC-SF* Activity-Specific Balance Confidence Scale Short Form, *PAS* Parkinson Activity Scale, *PDQ-39* Parkinson’s Disease Questionnaire-39, *SD* standard deviation, *CI* confidence interval, *ηp2* partial eta

^*^p < 0.05; **p < 0.01

## Discussion

In our study, statistically significant improvements were found in dynamic balance, postural stability, gait parameters, activity balance confidence, activity status, and quality of life in both LSVT® BIG and Progressive Structured Mobility Training groups. However, when comparing the groups, the improvements observed in dynamic balance, activity balance confidence, and activity status were in favor of the LSVT® BIG group. As the superiority of the LSVT® BIG group was only observed for self-rated scores (ABC-SF and PAS) and certain scale scores (Mini-BEST) that were scored by an unblinded assessor, these results need to be confirmed by a randomized controlled trial of high methodological quality.

When the effectiveness of exercise programs for PD patients is researched in the literature, it is seen that sustainable and task-specific program is suggested to improve gait speed, stride length, cadence and dynamic balance, and gait performance [6]. On the other hand, it is stated that balance exercises can improve balance control when performed together with other training modalities such as strengthening exercises or gait training in patients with PD. In addition, it has been reported that exercise and motor training programs including challenging balance training significantly improve activity-related balance performance [25]. But it is difficult to determine the superiority of any of the treatments due to the differences in duration, intensity of exercises, or outcome measures [4, 26]. Furthermore, there is limited evidence in the literature regarding the results of these suggested programs when applied with the telerehabilitation method synchronously [25]. So that the purpose of this study was to compare the effects of two different exercise protocols with the same dosage delivered through synchronous telerehabilitation on balance and mobility dysfunction in patients with PD.

Recent meta-analyses have indicated that rehabilitation programs can provide clinically significant benefits for gait and balance of patients with PD, and it was emphasized that the rehabilitation program should be “goal-based” (aiming to practice and learn certain activities in key areas), a set of practice variables (intensity, specificity, complexity) should be defined, and the program should be tailored to the characteristics of the patients [27]. Consistent with these meta-analyses, in our study, both groups which designed with same intensity but different content had improvements in both balance and gait parameters. While LSVT® BIG group received a program consisted of large amplitude tasks which were created based on the functional movements that the patient has difficulty in daily life activities, the other group received a progressive structured mobility training. Significant improvements which were obtained in favor of the LSVT® BIG group in Mini-BEST, activity status and activity balance confidence scores may be interpreted with the difference in the contents. It seems personalized goal-based training emphasized large amplitude led to more improvement in functional gain of PD patients in early terms. On the other hand, while personalized and structured contents seem to have similar effect on the gait speed and postural stability parameters, surprisingly, these improvements did not reflect to TUG results in both groups. As TUG is a time-dependent variable, it may not be sufficient to detect the difference in dynamic balance between these two different training programs in patients with early PD. As for the postural stability parameters, the difference between the groups cannot be shown because the device is evaluated at the most stable level. These conflict suggestions need to be proved with further research.

There are several studies in the literature that compare the effects of different exercise contexts in people with PD. Monticone et al. (2015) included seventy Parkinson’s patients with an H&Y stage of 2.5–4 in their study. Balance training based on activities including functional strategies for balance and gait was given to the experimental group, while passive and active joint mobilization, strengthening exercises, and standing or gait exercises were given to the control group through proprioceptive training. At the end of the 8-week treatment, the efficacy of the treatment was evaluated with the Berg Balance Scale and PDQ-39. The balance training group based on activities including functional strategies for balance and walking reported better results on balance and quality of life compared to the group performing only general mobilization, stretching, and resistance exercises [28]. Similarly, our study showed that significant improvements in terms of Mini-BEST and PAS are in favor of the LSVT® BIG group which included more functional strategies.

In 2018, Giardini et al. included 38 cases of PD diagnosed with H&Y stages 1–3 in their study, which aimed to compare two different balance training protocols (balance exercises on a moving platform and traditional balance exercises). In the balance exercises based on the Otago Exercise Program, the exercise sessions in both groups were applied from easy to difficult. While there was an improvement in the Mini-BEST results, gait speed, and TUG in both groups, no difference was found between the groups in their study [29]. In our study, synchronous telerehabilitation-based balance exercises of the same intensity had enhancing effects on balance and gait parameters, as well as level of activity level and quality of life in patients with mild to moderate PD. In favor of LSVT BIG group, there were statistically significant greater improvements in terms of Mini-BEST. Thus, task-oriented personalized exercise programs appear to be slightly more effective for dynamic balance.

When we reviewed the literature in terms of telerehabilitation delivery for PD population, recent research pointed out the importance of online physiotherapist supervision during the exercise programs performed by telerehabilitation. A randomized controlled study is conducted by Vasconcellos et al. with 28 patients with PD (H&Y stage 2–4); a protocol including telerehabilitation-based upper and lower extremity global exercises was applied to the control group (*n* = 14), while telerehabilitation-based strengthening exercises of trunk and pelvic floor was applied to the experimental group. The exercises were performed three times a day for 3 weeks in both groups. Physiotherapists followed the implementation of the exercises via e-mail or mobile messaging application by showing the booklets and videos prepared by the study team. Both groups were not found to be effective in improving the balance and gait of patients with PD [30]. It can be concluded that the lack of online synchronous supervision by the therapist may have had a negative impact on the patients’ performance during the interventions.

On the other hand, Seidler et al. used tango dance as a rehabilitation technique and investigated the effectiveness of tango dance with synchronized online sessions. The similar improvements were obtained in postural control results and in the changes in the group with face-to-face training and online training in the motor severity of the disease, and it was reported that there was no change in gait parameters in both groups. No superiority was found between the groups in terms of static and dynamic postural control results and motor severity of the disease [25]. It can be interpreted that telerehabilitation is a preferable option to face-to-face training in patients with PD when applied synchronously with a physiotherapist. In our study, it is believed that performing exercise programs synchronously with a physiotherapist during online telerehabilitation sessions caused improvements in both groups in terms of dynamic balance and gait parameters.

To our knowledge, it is the first randomized clinical trial to conduct the LSVT® BIG protocol based on synchronous telerehabilitation using a videoconferencing system and to compare the effects on balance and mobility dysfunction with another progressive structured mobility training program.

This study has some limitations that should be highlighted. Firstly, the results of our study were compared only in terms of pre- and post-treatment data; long-term results were not evaluated. Secondly, the assessor was not blinded to the groups. Finally, the wide age range may be an obstacle to the generalization of our results to specific age groups.

In conclusion, telerehabilitation-based LSVT® BIG protocol, which includes functional task-specific activities, is more beneficial for dynamic balance, activity balance confidence, and activity status in early stages of PD. On the other hand, a progressive structured mobility training can be as effective as LSVT® BIG treatment for postural stability, mobility and quality of life when were performed at the same intensity for early-stage PD. These findings need to be confirmed by more methodologically rigorous randomized controlled trial that minimize the risk of bias and monitor the long-term outcomes of interventions.

### Supplementary Information

Below is the link to the electronic supplementary material.

Supplementary file1 (DOCX 16 KB)

## Data Availability

There are no linked data sets for this paper. The data is confidential since the participants of this study were informed upon admission to the hospital that the data would remain confidential and would not be shared with third parties.
