# Supplementary material for: LSVT® BIG versus progressive structured mobility training through synchronous telerehabilitation in Parkinson’s disease: A randomized controlled trial
Source: Neurol Sci. 2024 Jan 25;45(7):3163–72. doi: 10.1007/s10072-024-07322-0 (PMC11176092; doi:10.1007/s10072-024-07322-0)
Supplement: Supplementary file 1 — Supplementary file1 (DOCX 16 KB) [file 10072_2024_7322_MOESM1_ESM.docx]

**Supplement 1. LSVT^®^ BIG Protocol**

| **Task** | **Exercise** | **Progression** |
| --- | --- | --- |
| **1) Maximal Daily Exercise** | Ex 1: 8 reps floor-to-ceiling reach and hold for 10 seconds  Ex 2: 8 reps reach right to left hold 10 seconds | Distal wrist and finger movements |
|  | Ex 3: step forward 16 reps  Ex 4: side step16 reps  Ex 5: side back 16 reps  Ex 6: reaching forward and rocking 20 reps  Ex 7: lying down and rocking 20 reps | Weight is added to the extremities, large strides with weights are continued. |
| **2) Functional Component Tasks** | 5 functional exercises based on sitting/standing basic complaints  (5 reps each) | Weights may be added to the extremities. The support surface can be changed by increasing the number of repetitions and the speed. |
| **3) Hierarchy Tasks** | Sequential application of functional components  1-3 tasks are selected (ex: wearing a jacket) | The number of repetitions is increased. By changing the order of functional tasks, they are made complex and difficult. |
| **4) BIG Walking** | Distance walking with a focus on large strides in reciprocal arm swings and upright posture | Walking on external floors, metronome, walking with reciprocal arm swing are practiced. |

**Supplement 2. Progressive Structured Mobility Training**

| **Week 1**  **(4 days a week / each 10 reps)** | **Week 2**  **(4 days a week / each 10 reps)** | **Week 3**  **(4 days a week / each 10 reps)** | **Week 4**  **(4 days a week / each 10 reps)** |
| --- | --- | --- | --- |
| Forward Step | Forward Step | Forward Step + Counting | Forward Step + open close arms |
| Side Step | Side Step | Side Step + Counting the Days of the Week | Side Step + open close arms |
| Backward Step | Backward Step | Backward Step + Counting the Days of the Week | Backward Step + open close arms |
| Side to side weight-bearing | Side to side weight-bearing | Standing and countdown + Counting down from 100 by fives | Standing and countdown + open close arms |
| Sit to Stand Exercise | Sit to Stand Exercise | Sit to Stand Exercise + Counting words starting with A | Sit to Stand Exercise + open close arms |
| Standing and countdown | 360 degree rotation around the chair | 360 degree rotation around the chair + Counting | Drawing “8” Around Two Chairs |
| Walking | Walking | Walking over obstacles | Walking by changing directions |
